# Loss-Optimized Design of Magnetic Devices

**DOI:** 10.3390/mi15060697

**Published:** 2024-05-24

**Authors:** Yuhu Zhao, Zhengfeng Ming, Chaofan Du

**Affiliations:** School of Mechanical and Electrical Engineering, Xidian University, Xi’an 710071, China; zhaoyuhu@stu.xidian.edu.cn (Y.Z.); 22041110415@stu.xidian.edu.cn (C.D.)

**Keywords:** finite element method, losses, magnetic devices, minimization, optimal design method

## Abstract

Maximizing efficiency, power density, and reliability stands as paramount objectives in the advancement of power electronic systems. Notably, the dimensions and losses of magnetic components emerge as primary constraints hindering the miniaturization of such systems. Researchers have increasingly focused on the design of loss minimization and size optimization of magnetic devices. In this paper, with the objective of minimizing the loss of magnetic devices, an optimal design method for the winding structure of devices is proposed based on the coupling relationship between the loss prediction model and the design variables. The method examines the decoupling conditions between the design variables and the loss model, deriving optimized design closure equations for the design variables. This approach furnishes a technical foundation for the miniaturized design of miniature apparatuses incorporating magnetic components, offering a straightforward and adaptable design methodology. The finite element method simulation results and experimental measurement data verify the accuracy of the prediction of the proposed method and the validity of the optimal design theory of device loss.

## 1. Introduction

Magnetic devices are essential components in modern life, serving a crucial role across various applications. In the realm of power electronics, devices like transformers and inductors facilitate the conversion of electrical energy [1] and provide electrical isolation, which is integral to power supply systems [2,3,4], micro-electro-mechanical systems [5,6], and electric vehicles [7,8]. Additionally, magnetic devices form the core of sensors [9,10] and implantable neural recording microsystems [11].

The application of magnetic devices improves the efficiency, performance, and reliability of the system. In recent years, wide bandwidth power devices, such as gallium nitride (GaN) and silicon carbide (SiC), have driven the high-frequency and miniaturization of systems [12]. However, the size, weight, and loss of magnetic devices occupy a major portion of the system and become the constraints for high frequency and miniaturization. Therefore, a large number of optimized designs have been proposed from three perspectives. First, newer core materials mean higher saturation flux density and permeability, resulting in smaller core size and loss [13,14]. Secondly, magnetic integration techniques are also favored by researchers [15,16,17,18]. However, this technique also improves the fabrication process and cost of magnetic devices. Third, the geometrical parameters of the magnetic core are associated with the losses of the device, and the optimal solution of the geometrical parameters of the core can be obtained to minimize the losses of the device [19,20,21]. The material and type of conductor, the winding structure, and the winding scheme determine the size and loss of the magnetic device [22,23,24,25].

In the design of magnetic devices, predicting device losses forms the basis for optimized design. These losses are typically divided into winding loss and core loss. Winding losses are influenced by the skin effect and proximity effect of the conductor, while core losses are directly linked to the material and size of the core, determined by the excitation source during operation. Various methods exist for predicting winding losses, including analytical and numerical approaches [26,27]. Notably, loss models for Litz wire devices [28] and 2D copper foil devices are continuously optimized [29,30,31]. Analytical prediction models for core losses encompass mathematical models, time-domain approximation models, loss separation models, and empirical models [1]. Additionally, the finite element method (FEM) can predict core losses [32,33]. Multi-physics field modeling and multi-objective optimization methods have also been employed to design magnetic devices based on losses, geometrical parameters, and device operation [18,34].

The multi-physics field model encompasses the circuit model, electromagnetic model, and thermal model of the system. Through finite element simulation, the coupling process between physical fields can be described more accurately, leading to a more precise device optimization scheme [35]. To expedite optimization, a common design strategy involves integrating multi-physics analysis models into the system optimization design workflow [2,36]. The multi-objective optimization method considers comprehensive parameters of system performance and refines the design objective function to achieve a tradeoff between size and loss, meeting specific design requirements. This method treats core loss and winding loss as two components and employs iterative optimization using algorithms such as the Particle Swarm Optimization Algorithm [37], the Genetic Algorithm [38,39], and the Pareto Optimization Algorithm [40], ultimately yielding a set of optimization results for the magnetic devices.

The aim of this paper is to propose a new design method for loss optimization of magnetic devices. Based on the analytical model of device losses, the method considers the coupling between winding losses and core losses and obtains a closed equation between winding geometric parameters, operating frequency, and total device losses. The method is faster to optimize and easier to use compared to algorithmic iterations. The method can be applied to the loss component of multi-physics field models and multi-objective optimization methods, thus reducing the computational and time costs of both methods.

The organization of this work consists of the following sections. The winding loss modeling of magnetic devices is described in Section 2. In Section 3, the optimal design of magnetic devices is modeled and extended for microdevice applications. The validation examples of the finite element method are given in Section 4. In Section 5, an experimentally validated design example is presented. In Section 6, conclusions are provided.

## 2. Winding Loss Modeling

**Figure 1:**
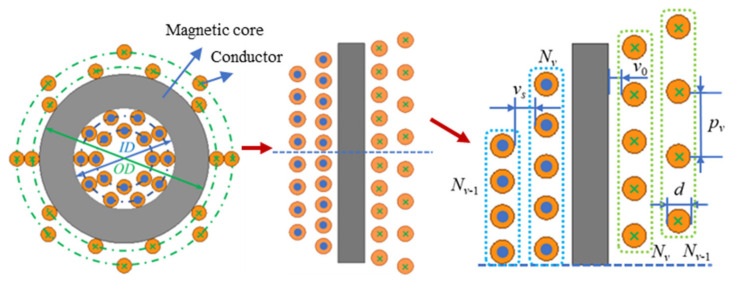
This is a two-dimensional diagram of a toroidal inductor. On the left is a two-dimensional cross-section of the inductor. In the center is a two-dimensional equivalent of the left-hand diagram, cut off and expanded along the core diameter. On the right is an enlarged view of the top half of the middle equivalent.

**Figure 2:**
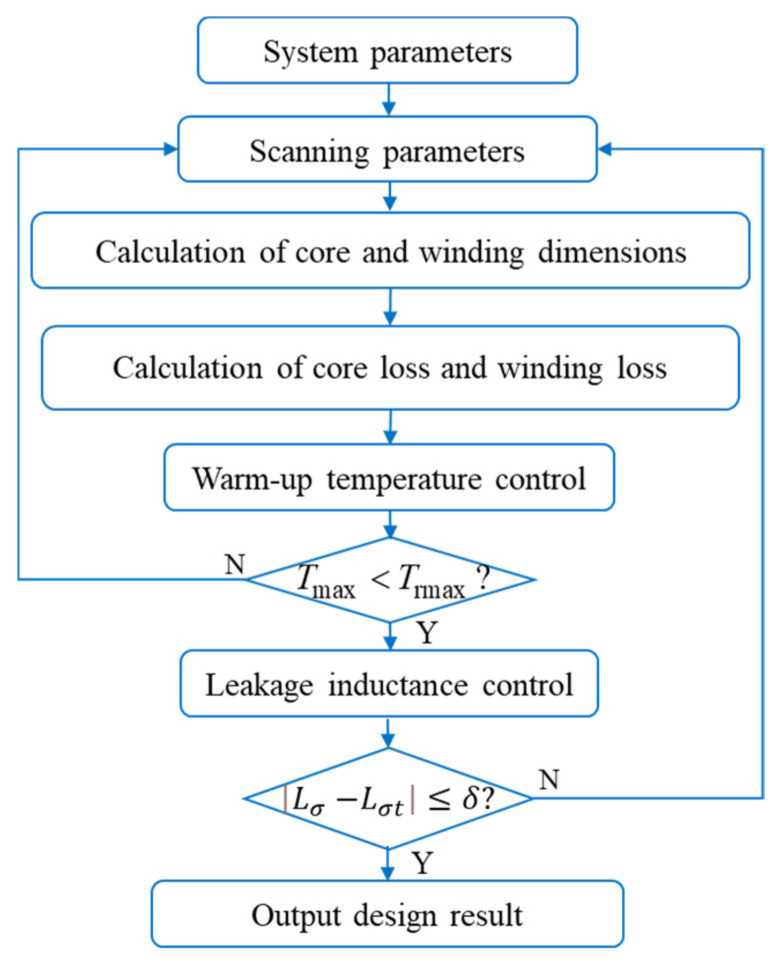
This is the design flowchart of the multi-objective optimization method for magnetic devices.

In this paper, a toroidal inductor, as depicted in Figure 1, is utilized as an example for winding loss analysis. The core has an inner diameter of *ID* and an outer diameter of *OD*. The solid round wire used for winding has a diameter of *d*, and the layer spacing of the windings is v_s_. It is assumed that each layer and each turn of the winding are uniformly wound on the core, with a sinusoidal current excitation of amplitude *I* on each turn of the core. The inner winding of the core is distributed correspondingly with the outer winding, and the total number of turns on the inner side and the total number of turns on the outer side are denoted by *n*. In this paper, the magnetic field distribution of one side of the winding is utilized to derive the analytical equation of the winding loss. In a toroidal inductor with multiple layers of windings, the number of turns in each layer of the winding may be different, and there are gaps between the conductors of each turn, so the porosity of each layer of the winding is not uniform. According to Ampere’s law, there is no magnetic field in these spaces [41]. In this paper, the magnetic field of the winding is calculated based on Ferreira’s method, taking into account the porosity factor of each winding layer. Assuming that the toroidal device has *m* layers of windings, the interlayer magnetic field inside and outside the *v*th layer of windings can be defined as follows:

(1) $${\dot{H}}_{inv}=\frac{\sum_{k=1}^{v-1}N_{k}\dot{I}\;}{2\pi r_{v}}\;\;\;\;\;\;\;{\dot{H}}_{outv}=\frac{\sum_{k=1}^{v}N_{k}\dot{I}}{2\pi r_{v}},$$

where the interlayer magnetic field on the inside of the *v*-th layer winding is ***H****_inv_*, and the interlayer magnetic field on the outside is ***H****_outv_*, as shown in Figure 2. *r_v_* is the radius of the *v*-th layer winding. *N_k_* is the number of turns of *k*-th layer winding and the amplitude of the excitation current in each turn winding is *I*.

(2) $$r_{v}=r_{ID}-0.1-(v-1)v_{s}-(v-0.5)d,$$

where *r_ID_* is the inner radius of the core.

The magnetic field strength ***H****_extv_* between the layers is known from the orthogonality of the skin and proximity effects.

(3) $${\dot{H}}_{extv}=0.5({\dot{H}}_{outv}+{\dot{H}}_{inv})=(2\sum_{k=1}^{v-1}N_{k}+N_{v})\dot{I}/4\pi r_{v}.$$

When using Ferreira’s method to calculate the loop magnetic field, the interlayer magnetic field may be overestimated. Therefore, we took into account the porosity parameter of the winding layers and corrected Ferreira’s method [34,41]. The modified Ferreira’s method is employed to calculate the AC losses of the solid round wire winding of the conventional structure (non-toroidal structure) [42]:

(4) $$P_{eac}=l\xi \left({\dot{I}}^{2}\psi_{1}(\xi )+2\pi^{2}d^{2}{\dot{H}}_{extv}^{2}\psi_{2}(\xi )\right)/\sqrt{2}\sigma \pi d^{2},$$

where *σ* is the equivalent conductivity of the winding. *l* is the length of the winding per turn. *ξ* is defined as follows:

(5) $$\xi =d/\sqrt{2}\delta =0.5d\sqrt{2\pi f\mu_{0}\sigma },$$

where *f* indicates the frequency of the excitation source, *μ*_0_ is the vacuum permeability. The equivalent skin depth is denoted by *δ*.

The skin effect coefficient and proximity effect coefficient of the modified Ferreira’s method are, respectively, expressed as follows [42]:

(6) $$\begin{array}{l} \psi_{1}(\xi )=\frac{ber_{0}(bei_{1}-ber_{1})-bei_{0}(bei_{1}+ber_{1})}{{bei_{1}}^{2}+{ber_{1}}^{2}}\;\;\\ \psi_{2}(\xi )=\frac{ber_{1}(bei_{2}-ber_{2})-bei_{1}(bei_{2}+ber_{2})}{{bei_{0}}^{2}+{ber_{0}}^{2}} \end{array}.$$

At low frequencies, the skin effect coefficient and the proximity effect coefficient can be equated to 2√2/*ξ* and √2*ξ*^3^/16, respectively.

*P_dc_* = ***I***^2^*l*/2*σπr*_0_^2^ indicates the DC loss of a unit length of a single-turn round wire winding. Combining (3) and (4), the average AC resistance coefficient of the toroidal inductor can be calculated by the modified Ferreira’s method as follows:

(7) $$\begin{array}{cc} F_{eac} & =\sum_{v=1}^{m}N_{v}P_{eac}/nP_{dc}\\ & =\xi [\psi_{1}(\xi )+0.125Dd^{2}n^{2}\psi_{2}(\xi )]/2\sqrt{2} \end{array},$$

(8) $$D=\sum_{v=1}^{m}\frac{\alpha_{v}{\left(2\sum_{k=1}^{v-1}\alpha_{k}+\alpha_{v}\right)}^{2}}{r_{v}^{2}}.$$

*α_v_* = *N_v_*/*n* denotes the percentage of turns of the *v*th winding layer of the winding in the total number of turns.

## 3. Winding Optimization Design

The modeling elements of an optimization problem in general form include design variables, an objective function, and constraints. Design variables can be categorized as continuous or discrete, with discrete variables having a higher optimization priority than continuous ones. When design variables are continuous, the objective function can be simplified using equational constraints and described by mutually independent variables. Constraints are further categorized as either equal or unequal constraints. Inequality constraints define boundary conditions for the design variables and the objective function, playing a crucial role in optimization design.

The optimal design of magnetic devices such as transformers and inductors follows the same rules of appeal. The multi-objective optimization method is a commonly used design method in magnetic devices and its flowchart is shown in Figure 2.

The optimized design solution that satisfies the constraints obtained based on the multi-objective optimization method contains multiple sets of solutions, which need to be enumerated and examined one by one to determine the optimal solution set. The method requires more computational expense. In this paper, the further optimal design of the device windings for the selected core is based on general optimal design rules, with the addition of constraints on the frequency variables and their objective functions, and parameterization of the equivalent model for the winding losses containing the frequency variables. The proposed method analyzes the decoupling conditions of the design variables and the device loss model and obtains the optimal design closure equations for the winding structure variables. Therefore, the optimization scheme is a general and simple winding optimization design method for magnetic devices.

### 3.1. Diameter of the Wire

Previous studies have employed methods such as the low-frequency equivalent AC resistance factor equation or fixed window factor and AC resistance factor method to determine the optimal wire diameter for minimizing winding loss. However, these approaches have not thoroughly explored the frequency range to which the optimal wire diameter equation applies. To address this gap, our paper proposes a novel approach to derive the wire diameter equation for minimizing winding loss. The proposed method decouples the control variables, treating the wire diameter as a variable while keeping the core size, winding turns, and arrangement fixed. Additionally, the proposed method considers the AC resistance factor and window utilization factor (porosity) across different frequency bands.

When the winding is operated at low frequencies, the bias derivatives of the AC resistance are taken to zero values according to (7).

(9) $$\frac{\partial R_{ac}}{\partial d}=\frac{\partial nF_{eac}R_{dc}}{\partial d}=\frac{4ln}{\sigma \pi }(\frac{-2}{d^{3}}+\frac{Dd^{3}n^{2}}{256\delta^{4}})=0\;\;\;\;\;\;\;\;\;\;\;\;\;\;\;\mathrm{for}\;\;\;\frac{d}{\delta }\leq 2,$$

where *R_dc_* = 4*l*/*σπd*^2^ is the DC resistance of the single-turn winding. Substituting (5) into (9) shows that the optimal diameter of the winding conductor *d_optLF_* is the following:

(10) $$d_{optLF}=2\sqrt[{6}]{8/{\left(\pi f\mu_{0}\sigma n\right)}^{2}D}.$$

By combining (7) and (10), it is clear that the optimum conductor diameter corresponds to an AC resistance coefficient of 1.5 and the winding losses are minimized at this time.

From (5) and (9), the maximum frequency of the applicable range of low frequency (LF) can be defined as follows:

(11) $$f_{LF}=4/\left(d^{2}\pi \mu_{0}\sigma \right).$$

Wojda defined the minimum frequency based on *F_ac_* = 1.05 in the optimized design of Litz wire winding. According to (5) and (6), the minimum frequency *f*_min_ for toroidal magnetic device winding optimization can be defined as follows.

(12) $$f_{\min}=16/\pi \mu_{0}\sigma d^{3}n\sqrt{5D}.$$

According to Equation (10) for the optimum diameter of the winding conductor, the value of *d_optLF_* increases sharply when *f* is reduced to a sufficiently low level. This will result in the maximum conductor diameter when the winding is filled (*F_ac_* is varied and porosity is fixed) is still less than *d_optLF_*, then the conductor diameter corresponding to the minimization of winding losses is the maximum allowed *d*_max_ = min(2π*r_v_*/*nα_v_*). When *d*_max_ = *d_optLF_*, the frequency of the winding is at the critical value of the winding optimized frequency *f_optp_*. From the closed equation of *d*_max_ and *d_optLF_*, it is known that the critical value of the winding optimized frequency is

(13) $$f_{optp}=\frac{2\sqrt{2}n^{2}\min{\left(\alpha_{v}/r_{v}\right)}^{3}}{\pi^{4}\sigma \mu_{0}\sqrt{D}}.$$

When *f*_min_ < *f* < *f_optp_*, the winding is filled with (*F_ac_* varying, porosity fixed) core, and the maximum conductor diameter allowed is the optimal diameter for winding optimization design; when *f_optp_* < *f* < *f_LF_*, the winding is not filled with (*F_ac_* fixed, porosity varying) core, and the optimal diameter for winding optimization design is *d_optLF_*.

When the winding works at high frequency (HF), the skin effect coefficient and the proximity effect coefficient are equivalent to the following:

(14) $$\psi_{1}{\left(\xi \right)}_{HF}\approx 1+y_{0}\;\;\;\;\;\;\;\;\psi_{2}{\left(\xi \right)}_{HF}\approx 1-y_{0},$$

where *y*_0_ is a constant. Set the error margin of the AC resistance factor high-frequency equivalent model as *e*_0_. Then, the equivalent model error is defined as follows:

(15) $$(R_{ac1}-R_{acHF})/R_{ac1}\times 100\%\leq e_{0},$$

where *R_ac_*_1_ denotes the AC resistance coefficient when both the skin effect coefficient and the proximity effect coefficient are 1, and it corresponds to an optimum conductor diameter of *d*_1_ = (8/*Dn*^2^)^0.5^. *R_acHF_* denotes the high-frequency equivalent AC resistance in this paper, corresponding to the optimal conductor diameter of *d_optHF_*. Combining (7), (9), and (14), *y*_0_ can be expressed as the following equation.

(16) $$y_{0}\geq (1-e_{0})d_{optHF}/d_{1}-1.$$

Then, according to (5), (6), (14), and (16), the minimum frequency *f_HF_* applicable to the high-frequency equivalent model of the winding can be obtained.

Combining (5), (9) and (23), the derivative for the AC resistance is obtained as follows:

(17) $$\frac{\partial R_{ac}}{\partial d}=\frac{nl}{\sigma \pi \delta }(\frac{-1-y_{0}}{d^{2}}+\frac{Dn^{2}(1-y_{0})}{8})=0\;\;\;\;.$$

By simplifying (17), the optimal conductor diameter *d_optHF_* for HF can be expressed as follows:

(18) $$d_{optHF}=2\sqrt{2(1+y_{0})/Dn^{2}(1-y_{0})}.$$

From (16) and (18), the value of *y*_0_ is (1 − (1 − *e*_0_)^2^)^0.5^. When the size of the core, turns of winding, and arrangement are constant, *d_optHF_* is a constant and does not vary with *f*. Combining (7), (14), and (18), the AC resistance factor for minimizing the loss in the HF winding can be defined as follows:

(19) $$F_{optHF}=(1+y_{0})\sqrt{2\pi f\mu_{0}\sigma (1+y_{0})/Dn^{2}(1-y_{0})}.$$

The AC resistance coefficient of the optimized design of the high-frequency winding varies with *f*, which is different from that of the low frequency.

When *f_LF_* < *f* < *f_HF_*, the winding is not filled (*F_ac_* changes, porosity changes) with core, the analytical equation of *R_ac_* versus *d* can be obtained from (7) and (9), and the minimum value of AC resistance is defined as min (*d*, *R_ac_*), then the conductor diameter corresponding to the minimum value of AC resistance is the optimal conductor diameter *d_optMF_*; when *f* > *f_HF_*, the winding is not filled (*F_ac_* changes, porosity changes) with core, and the optimal diameter of the winding optimized design is *d_optHF_*.

It should be noted that when the maximum conductor diameter allowed in the winding is less than the optimal diameter of the winding conductor in any frequency band, the maximum conductor diameter is taken in the winding optimization design.

### 3.2. Number of Turns

The number of turns in a winding not only impacts the eddy current losses but also alters the AC flux, thus affecting the core losses. Various analytical methods have been proposed to explore the relationship between AC flux and total losses in magnetic devices, particularly considering the influence of winding turns on total device losses at low frequencies. However, these methods typically focus on low-frequency optimization and impose fixed conditions such as the AC resistance factor, limiting the approach to device optimization design. To achieve the minimum total loss in a magnetic device, it is common to calculate the optimal loss ratio between winding eddy current loss and core loss. In this paper, the optimal loss ratio for different AC resistance coefficients and window utilization coefficients (porosity) are considered in frequency bands for the optimal design of winding turns, and the other parameters of the device are fixed.

For multi-winding magnetic devices, the analytical relationship between the number of turns and the AC flux is obtained from Faraday’s law [43].

(20) $$B_{ac}=V_{\mathrm{rms}}/K_{s}fnA_{c},$$

where *B_ac_* is the AC magnetic induction strength, *V*_rms_ is the rms value of the impressed voltage of winding, *K_s_* is a waveform factor, and *A_c_* is the core cross-sectional area.

The core loss of a magnetic device can be expressed as follows [43].

(21) $$P_{Fe}=C_{m}f^{\alpha }B_{ac}^{\beta }V_{c}=C_{m}V_{c}f^{\alpha }{\left(\frac{V_{\mathrm{rms}}}{K_{s}fnA_{c}}\right)}^{\beta }=\frac{K_{1}}{n^{\beta }}.$$

The eddy current loss in the winding of a magnetic device can be defined as follows:

(22) $$P_{Cu}=nF_{eac}R_{dc}I_{\mathrm{rms}}^{2}=4nlI_{\mathrm{rms}}^{2}F_{eac}/\sigma \pi d^{2}.$$

According to the conclusion of Section 3.1, when *f_optp_* < *f* < *f_LF_*, the winding is not filled (*F_ac_* is fixed and porosity varies) with the core, and the optimal diameter of the winding conductor corresponds to an AC resistance factor of 1.5. Substituting Equation (10) into Equation (22), the winding losses at low frequencies can be expressed by the following equation.

(23) $$P_{CuLF}=0.75ln^{5/3}I_{\mathrm{rms}}^{2}\sqrt[{3}]{f^{2}\mu_{0}^{2}D/\sigma \pi }=K_{2}n^{5/3}.$$

In order to minimize the total loss of the magnetic device, let the partial derivative of the total loss be equal to 0. The optimal loss ratio of winding eddy current loss and core loss and their corresponding number of turns can be obtained.

(24) $$\frac{P_{CuLF}}{P_{Fe}}=\frac{3\beta }{5}\;\;\;\;\;\;\;\;\;\;\;\;\;\;\;n_{optLF}={\left(\frac{3\beta K_{1}}{5K_{2}}\right)}^{3/\left(5+3\beta \right)}.$$

When *f*_min_ < *f* < *f_optp_*, the winding fills (*F_ac_* changes, porosity is fixed) the core, and the maximum conductor diameter allowed is the optimal diameter for the optimal design of the winding; then, the eddy current loss of the winding at this time can be defined as follows:

(25) $$\begin{array}{cc} P_{CuLF} & =lI_{\mathrm{rms}}^{2}(\frac{\min{\left(\alpha_{v}/r_{v}\right)}^{2}n^{3}}{\sigma \pi^{3}}+\frac{\pi^{3}D\min{\left(r_{v}/\alpha_{v}\right)}^{4}}{16n\sigma \delta^{4}})\\ & =K_{21}n^{3}+K_{22}/n \end{array}.$$

Taking the same approach of solving for partial derivatives, the equation for the optimal number of turns can be obtained.

(26) $$3K_{21}n^{3}-\frac{K_{22}}{n}=\frac{K_{1}\beta }{n^{\beta }}.$$

Based on the conclusion in Section 3.1, when *f_LF_* < *f* < *f_HF_*, the minimum value of AC resistance is min (*d*, *R_ac_*) when the winding is not filled (*F_ac_* changes, porosity changes) with the core, then the conductor diameter corresponding to min (*d*, *R_ac_*) is the optimal conductor diameter *d_optMF_*. All parameters are fixed except conductor diameter and the number of turns, and a set of n values is set to fit the analytical equation between *n* and *d_optMF_*. The analytical equations for the winding losses are obtained by substituting the fitted equations into (7) and (22), and then the partial derivatives of the total losses are combined with (21) to obtain the optimum loss rate and number of turns for optimum winding design.

When *f* > *f_HF_*, the winding is not filled (*F_ac_* changes, porosity changes) with the core, and the optimal diameter of the winding optimized design is *d_optHF_*. According to (18), (19), and (22), the eddy current loss of the winding can be expressed as follows.

(27) $$P_{CuHF}=ln^{2}I_{\mathrm{rms}}^{2}(1-e_{0})\sqrt{\mu_{0}fD/2\sigma \pi }=K_{23}n^{2}.$$

The total loss of the winding can be obtained from the sum of (21) and (27). The partial derivatives of the total loss with respect to the number of turns are used to compute the poles. Then, the optimal loss ratio and the optimal number of turns of the winding for high-frequency magnetic devices are defined as follows.

(28) $$\frac{P_{CuLF}}{P_{Fe}}=\frac{\beta }{2}\;\;\;\;\;\;\;\;\;\;\;\;\;\;\;n_{optLF}={\left(\frac{\beta K_{1}}{2K_{23}}\right)}^{1/\left(2+\beta \right)}.$$

### 3.3. Winding Arrangement

After determining the conductor diameter and number of turns, the winding arrangement also influences the loss of the magnetic device. During the winding optimization design process, reducing the number of winding layers can decrease winding loss. This paper analyzes the effect of winding arrangement on loss using a three-layer winding as an example. When the sum of the turns ratio of winding layers is 1, Equation (8) is simplified as follows:

(29) $$\begin{array}{l} D_{3}=\sum_{v=1}^{3}(\alpha_{v}{\left(2\sum_{k=1}^{v-1}\alpha_{k}+\alpha_{v}\right)}^{2}/r_{v}^{2})\\ \geq \left(\alpha_{1}^{3}+{\left(2\alpha_{1}+\alpha_{2}\right)}^{2}\alpha_{2}+{\left(2\alpha_{1}+2\alpha_{2}+\alpha_{3}\right)}^{2}\alpha_{3}\right)/r_{3}^{2}\\ =\left((\alpha_{2}-1)\alpha_{1}^{2}+{\left(1-\alpha_{2}\right)}^{2}\alpha_{1}+\alpha_{2}-\alpha_{2}^{2}+1\right)/r_{3}^{2} \end{array}.$$

Assuming *α*_2_ is a constant and *α*_1_ is a variable, the maximum value of *D*_3_ is *α*_10_ = 0.5(1 − *α*_2_). And the inner winding turns of the toroidal magnetic device are more than the outer layer, it is known that *α*_1_ ≤ *α*_2_ ≤ *α*_3_, so the value of *α*_10_ can be defined as follows:

(30) $$\alpha_{10}=0.5(1-\alpha_{2})=0.5(\alpha_{3}+\alpha_{1})\geq \alpha_{1}.$$

From (30), it is obvious that the value of *α*_1_ is taken to the left of *α*_10_, and at this time the value of *D*_3_ increases with the increase in *α*_1_. According to (7), the winding loss increases. In the winding optimization design, the windings are preferentially arranged in the inner layer.

### 3.4. Optimal Design Model

**Figure 3:**
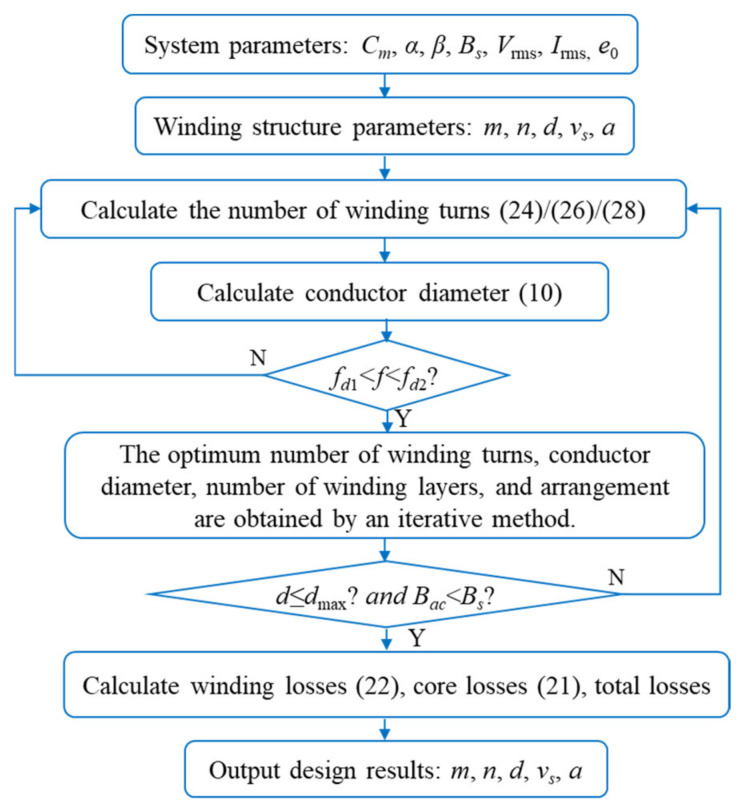
Flowchart for the optimized design of the winding structure of magnetic devices.

The objective of the optimization design model is to determine the winding structure of the magnetic device in order to minimize total losses. The core parameters *C_m_*, *α*, and *β* are determined by the parameter manual of the selected core. Following general optimal design rules, the design variables of the magnetic device are determined: excitation parameters (*f*, *K_v_*, *V*_rms_, *I*_rms_), winding geometrical parameters (*m*, *v_s_*, *d*, *n*, α), and error margin parameter *e*_0_. Where *m*, *n*, *a*, and *d* are discrete variables to be optimized preferentially. The objective function is the total loss of the magnetic device. The constraints include ensuring that *B_ac_* is less than the saturation magnetic induction and that the winding cross-sectional area is less than the maximum allowable window area. Based on the aforementioned design basis properties, an optimized design flow for the winding structure of the magnetic device is proposed, as shown in Figure 3.

According to Figure 3, an example of computational flow is presented. System parameters were determined for the given setup, with a frequency (*f*) of 100 kHz, and coefficients (*C_m_*), (*α*), and (*β*) of 62.22, 1.561, and 2.103 respectively. The core cross-sectional area (*A_c_*) and volume (*V_c_*) were measured at actual values of 33.87 mm^2^ and 1959.5 mm^3^, respectively.

Voltage (*V*_rms_) and current (*I*_rms_) within the device were recorded at 80 V and 0.8 A respectively.

Setting *m* = 1, we utilize equation (28) to determine the value of *n_opt_*. The value of *n_opt_* is determined to be 102.6; however, since the *n_opt_* must be an integer, it is rounded up to 103. Substituting *n_opt_* = 103 into equation (10), then *d_opt_* = 0.426 mm. At this juncture, it is observed that *f_opt_* > *f_LF_* and *d*_max_ = 0.384 mm.

Since with *m* = 1, neither the frequency nor the conductor diameter condition is met, we proceed by setting *m* to 2 and *a* to 8/9, based on the calculated optimal number of turns and conductor diameter. This process is then repeated.

The value of *n_opt_* is determined to be 101.4. Given that *n_opt_* must be an integer, the optimal number of turns is rounded to 101. Substituting *n_opt_* =101 into equation (10), then *d_opt_* = 0.423 mm. Consequently, the selected conductor type is identified as AWG26, with the closest available conductor diameter (*d_opt_*) of 0.404 mm.

Substituting the value of *d_opt_* into (11) and (13), we determine the upper and lower frequency limits to be *f_LF_* = 108.6 kHz and *f_opt_* = 85.34 kHz, respectively. Given that the excitation frequency (*f*) falls within this frequency range, it complies with the frequency conditions of the LF method. The layer spacing (*v_s_*) is set at 0.1 mm, and *d*_max_ = 0.44 mm. Additionally, the magnetic flux density (*B_ac_*) is calculated to be 0.0527 T, while the maximum allowable magnetic flux density (*B_s_*) is 0.4 T. Both the conductor diameter and magnetic flux density conditions are thus satisfied.

Using equation (22), we calculate the winding loss (*P_Cu_*) to be 0.45 W, and the core loss (*P_Fe_*) to be 0.331 W, resulting in a total loss (*P_total_*) of 0.781 W. With a winding comprising 2 layers (*m* = 2), an optimal number of turns (*n_opt_*) of 101, and an optimal conductor diameter (*d_opt_*) of 0.404 mm, along with a layer spacing (*v_s_*) of 0.1 mm and *a* = 0.89, these parameters collectively inform the system’s performance characteristics.

### 3.5. Extension of Theory and Miniaturized Systems

In the second and third sections, the theory of loss optimization design is explained using the toroidal magnetic device with solid wire. However, in practice, the shape of the magnetic device, the type of conductor, and the excitation conditions vary. Therefore, it is necessary to further extend the design theory and apply it to miniaturized systems.

The common shapes of magnetic devices are categorized as toroidal and rectangle. In the winding loss model, the porosity of the toroidal device varies with the number of winding layers, while the porosity of the rectangular device remains constant. This leads to variations in magnetic fields between the winding layers. The interlayer magnetic field of a rectangular device can be expressed as follows:

(31) $${\dot{H}}_{inv}=\frac{\sum_{k=1}^{v-1}N_{k}\dot{I}\;}{h}=\frac{(v-1)N_{0}\dot{I}}{h}\;\;\;\;\;\;\;\;\;\;\;\;{\dot{H}}_{outv}=\frac{\sum_{k=1}^{v}N_{k}\dot{I}}{h}=\frac{vN_{0}\dot{I}}{h},$$

where *h* is the window height and *N*_0_ is the number of turns per layer of windings. By replacing (1) with (31), the proposed method can be applied to the optimized design of rectangular devices.

The types of conductors are categorized as solid wire, copper foil, and Litz wire. The solid wire and copper foil can be converted to each other by the equivalent area method.

(32) $$b^{2}=\pi d^{2}/4,$$

where b is the width of the copper foil and d is the diameter of the solid wire. According to (7) and (32), by converting *d* to *b*, the proposed method can be generalized to the optimal design of magnetic devices wound with copper foil. In most cases, the analytical copper loss model considering air gap and fringing effects of copper foil magnetic devices and PCB can also be applied to the above optimization design method. The two-dimensional AC resistance model is defined as follows [30]:

(33) $$\begin{array}{l} R_{\mathrm{ac},n}=R_{\mathrm{skin},n}+R_{\mathrm{prox},n}+R_{\mathrm{fring},n}\\ =R_{\mathrm{dc},n}\frac{1}{2}S_{\mathrm{ESC},0}+R_{\mathrm{dc},n}\frac{{\left(2n-1\right)}^{2}}{2}S_{\mathrm{OSC},0}+R_{\mathrm{dc},n}N^{2}V_{n} \end{array}.$$

The planar helical coils of implantable neural recording microsystems comprise PCB and copper foil magnetic devices, and the extended theory can optimize their design effectively. Its AC resistance model can be incorporated into the proposed optimization method, and the model is presented below [11,44]:

(34) $$R_{AC}=R_{DC}\xi (\Delta_{1}+\frac{\Delta_{2}\cdot {\left(Nw\right)}^{2}}{3\cdot {\left[(Nw)+(N-1)s\right]}^{2}}).$$

The MEMS quadruple electromagnetic transformer, integrated into a single chip, can also benefit from optimal design using the extended theory. Its resistance model can be defined as follows [3]:

(35) $$R=R_{w}(N)+2\pi f(\frac{\mu^{\prime \prime }}{\mu^{\prime }})\Delta L(N^{2}).$$

**Figure 4:**
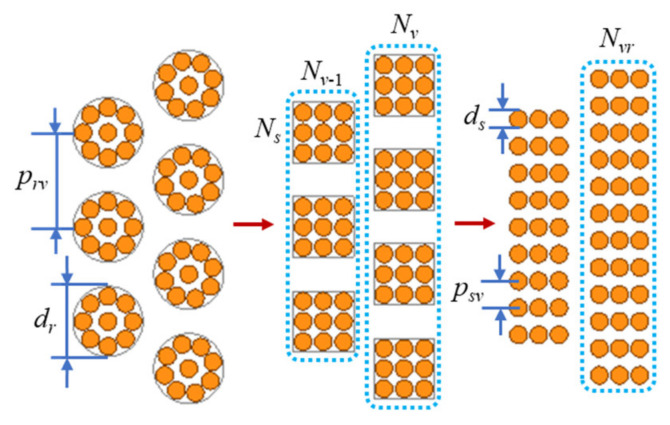
Process diagram of Litz wire equivalent to solid wire.

The loss analytical methods for the windings with Litz wire are generally based on Dowell’s method and Ferreira’s method. The Litz wire can be equated to solid wire as shown in Figure 4.

The interlayer magnetic field of Litz wire windings can be defined as follows:

(36) $${\dot{H}}_{inv}=\;\sum_{k=1}^{v-1}N_{k}N_{s}{\dot{I}}_{s}/2\pi r_{v}\;\;\;\;\;\;{\dot{H}}_{outv}=\sum_{k=1}^{v}N_{k}N_{s}{\dot{I}}_{s}/2\pi r_{v},$$

where *I_s_* is the current in each strand of conductor and *N_s_* is the number of strands of conductor in a single turn of Litz wire. In (2), *d* = *d_r_*, and in (4) and (5), *d* = *d_s_*. Then, the AC resistance coefficient of magnetic devices with Litz wire can be expressed as follows:

(37) $$F_{esac}=\xi_{s}[\psi_{1}(\xi_{s})+0.125Dd_{s}^{2}n^{2}N_{s}^{2}\psi_{2}(\xi_{s})]/2\sqrt{2}.$$

Replacing (7) with (37), the optimal design method for real conductors can be extended to the Litz wire. The improved analytical model of Litz wire is also applicable to the method. For example, the analytical model for the copper loss of Litz wire with multiple levels of twisting is shown below [31].

(38) $$\begin{array}{l} R_{L}=\frac{m\rho }{\pi \alpha_{s}^{2}n_{\mathrm{total}}}F\left(\gamma_{s}\right)F\left(\gamma_{b}\right)+\frac{4\pi \rho n_{\mathrm{total}}K\left(\gamma_{s}\right)}{8\pi^{2}\alpha_{L}^{2}}\left(\frac{4m^{3}}{3}-\frac{13m}{6}+\frac{11}{6m}\right)\;\;(\mathrm{LF})\\ G_{L}=4\pi \rho n_{\mathrm{total}}K\left(\gamma_{s}\right)\left(\frac{3m}{4}+\frac{1}{4m}\right)\;\;(\mathrm{HF}) \end{array}.$$

The core loss models are applicable to different operating conditions. Due to the large amount of data required, high computational cost, and harsh application conditions, mathematical models and time-domain approximation models are not commonly used in the design of magnetic devices. The loss separation model divides the core loss into three parts [1].

(39) $$P_{core}=k_{h}fB_{s}^{x}+k_{eddy}f^{2}B_{s}^{2}+k_{anom}f^{1.5}B_{s}^{1.5},$$

where *k_h_*, *k_eddy_*, and *k_anom_* are constants. The model can calculate the core loss for square wave excitation with DC bias. Equation (39) is consistent with the variables and functions of (21) and can be used for the optimal design of magnetic devices under square wave excitation.

Empirical models are commonly used for core loss prediction. Among the typical models applicable to arbitrary non-sinusoidal excitation are the MSE model and the iGSE model. Their models can be expressed in the following general form.

(40) $$P_{core}=kC_{m}f^{\alpha }B_{ac}^{\beta }V_{c},$$

where *k* is a constant related to the actual waveform parameters. By replacing (21) with (40), the proposed design method can be extended to the optimization of magnetic devices with arbitrary non-sinusoidal excitation.

Common non-sinusoidal excitations encountered in converters include square, rectangular, and triangular waves. This paper investigates the core loss under non-sinusoidal excitation, using triangular wave excitation in a buck converter as an illustrative example. The core loss of the device, computed using the MSE method, is expressed by the following equation [45].

(41) $$P_{c}{|}_{\mathrm{MSE}}=\left(\begin{array}{c} C_{m}\cdot f_{\mathrm{eq}}^{\alpha -1}\cdot B_{m}^{\beta } \end{array}\right)\cdot f,$$

where the equivalent frequency *f_eq_* can be expressed as follows:

(42) $$f_{\mathrm{eq}}=\frac{2}{\Delta B^{2}\pi^{2}}\int_{0}^{T}{\left(\frac{dB}{dt}\right)}^{2}dt.$$

The MSE method mainly involves the integration of the rate of change of magnetic induction (*dB*/*dt*). A direct relationship exists between *dB*/*dt* and voltage:

(43) u(t)=dψ(t)/dt=nSdB(t)/dt,

where *ψ* represents the magnetic flux, and *S* denotes the effective magnetic circuit area. The functional expression describing a triangular wave over one cycle is as follows:

(44) $$u(t)=\left\{\begin{array}{c} \frac{4U_{m}}{T}t,\;\;\;\;\;\;\;\;\;\;\;\;\;\;0<t\leq \frac{1}{4}T\\ -\frac{4U_{m}}{T}(t-\frac{T}{2}),\;\;\frac{1}{4}T<t\leq \frac{3}{4}T\\ \frac{4U_{m}}{T}(t-T),\;\;\;\;\;\frac{3}{4}T<t\leq T \end{array}\right.,$$

where the voltage amplitude *U_m_* is given by 8*fnB_m_S*. Together with Equations (41)–(44), the core loss of the triangular wave can be expressed as the following equation.

(45) $$P_{Fe}={\left(32/3\pi^{2}\right)}^{\alpha -1}C_{m}f^{\alpha }B_{m}^{\beta }V_{c}.$$

The calculation of winding losses for triangular waves follows the same methodology as for sinusoidal excitation. By substituting Equation (21) with (45), the proposed method can be extended to optimize the design of devices for triangular wave excitation.

Besides the impact of the non-sinusoidal nature of the excitation waveform on losses, the presence of DC bias also influences core losses. In boost and buck converter applications, the excitation passing through the magnetic device comprises both AC and DC components. DC bias solely affects the hysteresis loss, leaving the eddy current loss unaffected, as it is solely contingent upon the rate of change of magnetic flux density [46]. The core loss model under DC bias is expressed by the following equation [46]:

(46) $$\begin{array}{cc} P_{Fe} & =P_{h}(\Delta B)V_{c}=P_{h0}\varepsilon (\Delta B)V_{c}\\ & =C_{m}f^{\alpha }B_{ac}^{\beta }(1+k_{dc}\Delta B^{k_{\alpha }}+k_{l}\Delta B^{2})V_{c} \end{array},$$

where the peak-to-peak value of the magnetic induction intensity Δ*B* in one magnetization cycle *T* is given by *B*_max_ − *B*_min_, where *B*_max_ and *B*_min_ represent the maximum and minimum values of the magnetic induction intensity, respectively. Constants *k_dc_*, *k_α_*, and *k_l_* can be determined by fitting the test data of the core loss.

The winding loss *P_Cu_* under DC bias is expressed as *I_dc_*^2^*R_dc_* + *I_ac_*^2^*R_ac_*. By substituting Equation (21) with (46), the proposed method can be extended to optimize the design of devices considering DC bias.

## 4. Finite Element Method Case

### 4.1. Diameter of the Wire Case

**Figure 5:**
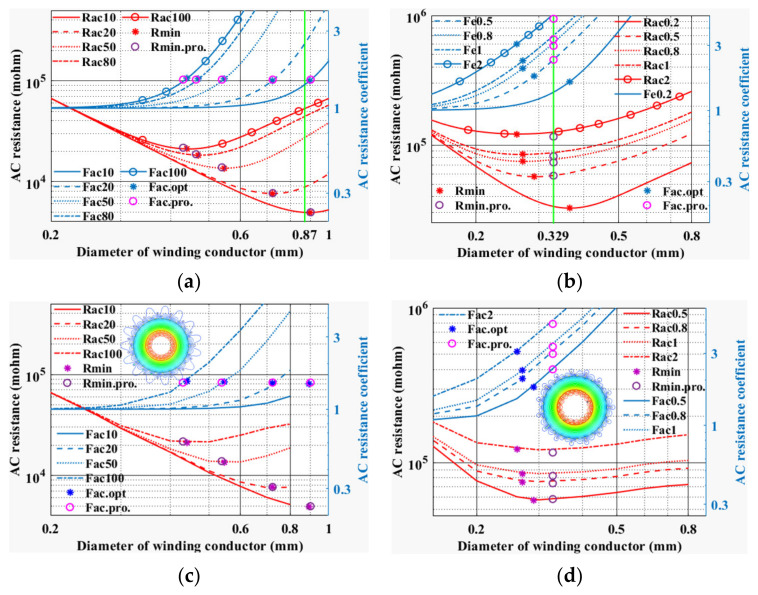
Result plots for the optimum conductor diameter of the windings: (**a**) plot of comparison results between analytical method and LF equivalent method, (**b**) plot of comparison results between analytical method and HF equivalent method, (**c**) plot of comparison results between FEM and LF equivalent method, (**d**) plot of comparison results between FEM and HF equivalent method.

In this section, the optimal conductor diameter designs for low-frequency bands (10 kHz, 20 kHz, 50 kHz, 100 kHz) and high-frequency bands (0.5 MHz, 0.8 MHz, 1 MHz, 2 MHz) are analyzed, and a set of FEM models with device parameters shown in Table 1 are constructed. The analytical results and simulation results of the optimal conductor diameter of the winding are shown in Figure 5.

Figure 5 depicts the design results of the optimal conductor diameter of the winding at different frequencies. *R_acf_* denotes the AC resistance of the winding at frequency *f*, and *F_acf_* denotes the AC resistance coefficient at frequency *f*. *R*_min_ is the minimum resistance of the analytical method, and its corresponding AC resistance coefficient is *F_ac_*_.*opt*._. The minimum resistance calculated by the proposed approximate method is defined as *R*_min.*pro*._ and its corresponding AC resistance factor is *F_ac_*_.*pro*._. From Figure 5a, when *f* is 10 kHz, *d_op_*_t_ is greater than the maximum allowable diameter of the winding 0.87, and then *d_opt_* is taken as 0.87. When the frequency increases, the AC resistance coefficient of the winding is about 1.5 and the optimal conductor diameter decreases. The optimal conductor diameter calculated by the LF equivalent method is consistent with that of the analytical method. And as can be seen in Figure 5c, the optimal conductor diameter simulated by FEM is close to that predicted by the LF equivalent method. In Figure 5b, the optimal conductor diameter predicted by the HF equivalent method is a constant of 0.329, which corresponds to an AC resistance factor that increases with frequency. The minimum AC resistance value designed by the HF equivalent method is similar to that of the analytical method. As shown in Figure 5d, the optimal conductor diameter and the minimum AC resistance predicted by the HF approximation method are the same as those simulated by the FEM. According to Figure 5, it is obvious that the FEM verifies the effectiveness of the proposed method in the optimal design of the conductor diameter of magnetic devices.

### 4.2. Number of Turns Case

**Figure 6:**
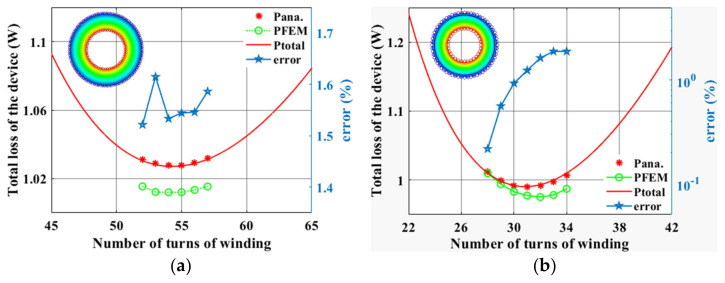
Comparative results of optimum turns design of windings and finite element method: (**a**) plot of results and errors at 100 kHz, (**b**) plot of results and errors at 800 kHz.

In this section, the optimized design of the number of turns for low-frequency 100 kHz and high-frequency 800 kHz are discussed, respectively. And two sets of magnetic device models were constructed using FEM, and the device parameters are shown in Table 2. The magnetic core is MPP55310 with material parameters: *C_m_* = 62.22, *α* = 1.561, *β* = 2.103. The core cross-sectional area and volume are actual values: *A_c_* = 33.87 mm^2^, *V_c_* = 1959.5 mm^3^. *V*_rms_ and *I*_rms_ in the device are 50 V and 1.5 A, respectively. The analytical results and simulation results of the optimal number of turns of the windings are shown in Figure 6.

Figure 6 depicts the design of the optimal number of turns for different frequencies. The total loss of the device designed by the approximation method proposed in this paper is defined as *P_ana_*. *P_FEM_* denotes the total loss simulated by FEM. *P_total_* is the total loss of the device estimated by the analytical method and determined by (21) and (22). *error* denotes the error between the total loss predicted by the approximation method and FEM. 

In Figure 6a, the optimal number of turns predicted by all three methods is 55 turns at a frequency of 100 kHz, and the total loss of their corresponding devices is the minimum. The maximum error between the low-frequency approximation method and FEM is 1.6%. According to Figure 6b, the optimal number of turns predicted by the analytical method is between 31 and 32 at a frequency of 800 kHz. The optimal number of turns estimated by FEM is 32 turns, and their corresponding minimum total device losses are very close, which is consistent with the theoretical results. The total loss obtained by the analytical method is higher than the simulation method because the analytical method overestimates the winding loss of the device. The maximum error between the high-frequency approximation method and FEM is 1.9%. This design error is within the allowed range, and FEM verifies the validity of the optimal design method for the winding turns.

## 5. Experimental Analysis and Discussion

Winding design significantly influences the performance of magnetic devices. Factors such as the number of turns, conductor diameter, number of layers, and arrangement of the windings all impact the loss of the magnetic device. To validate the effectiveness of the proposed design approach, the frequency response of these coupling factors is taken into account. A set of low-frequency (100 kHz) toroidal magnetic core device samples is designed according to the flow depicted in Figure 3, with the device parameters listed in Table 3. The core model of the device samples is MPP55310.

**Figure 7:**
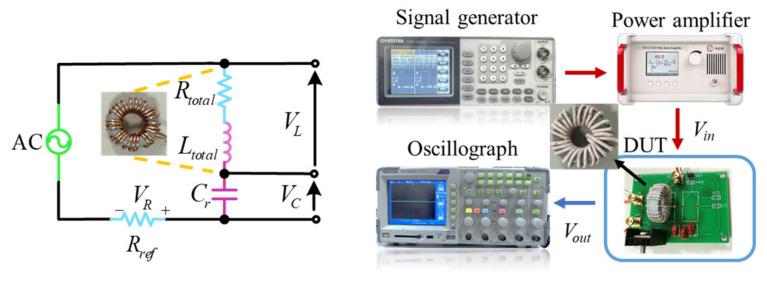
Circuit schematic and physical diagram of magnetic device loss measurement.

The measurement of winding loss in magnetic devices is generally categorized into the impedance analysis method and the power measurement method. The impedance analysis method does not consider the effect of actual operating conditions on winding loss and is suitable for measuring winding loss with sinusoidal excitation [21]. While the power measurement method considers the actual operating conditions of the magnetic device, its measurement accuracy depends on impedance angle measurement. Commonly used measurement methods for magnetic core loss include the DC power method and the double-winding AC power method. Determining other circuit losses in the DC power method is more complex. The double-winding AC power method is less accurate in measuring the core loss of high impedance angle and ultra-high frequency magnetic devices [47]. To address this issue, Mu proposed the method of series capacitance compensation, which reduces the phase angle of the measured voltage and current, thereby improving measurement accuracy. This method is only applicable to sinusoidal excitation, and its accuracy depends on whether the capacitor can completely compensate for the inductive reactive power of the measured device. In this paper, the total power of the magnetic device is measured using the method of non-complete capacitive compensation [48], and the measurement circuit is shown in Figure 7.

As shown in the circuit schematic in Figure 7, the incomplete capacitive compensation method requires the measurement of the voltage values of the inductor, capacitor, and resistor. A virtual voltage *V*_3_ representing the compensation effect is created, and then the voltage is defined as follows:

(47) $$V_{3}=V_{L}+\frac{1}{k}V_{C}\;\;\;\;\;\;\;\;\;\;\;\;k=\frac{V_{C}}{V_{L}\cdot \sin\varphi_{2}},$$

where *φ*_2_ is the phase angle between the inductor voltage *V_L_* and the capacitor voltage *V_C_*. The current limiting resistance is denoted by *R_ref_*. The current of the loop can be defined as *I* = *V_R_*/*R_ref_*. Then, the total loss of magnetic devices can be expressed as follows:

(48) $$P_{total}=\frac{f}{R_{ref}}\int_{0}^{T}V_{L}V_{R}dt+\frac{1}{k}\frac{f}{R_{ref}}\int_{0}^{T}V_{C}V_{R}dt=\frac{1}{T}\int_{0}^{T}V_{3}Idt,$$

where *T* denotes the period.

**Figure 8:**
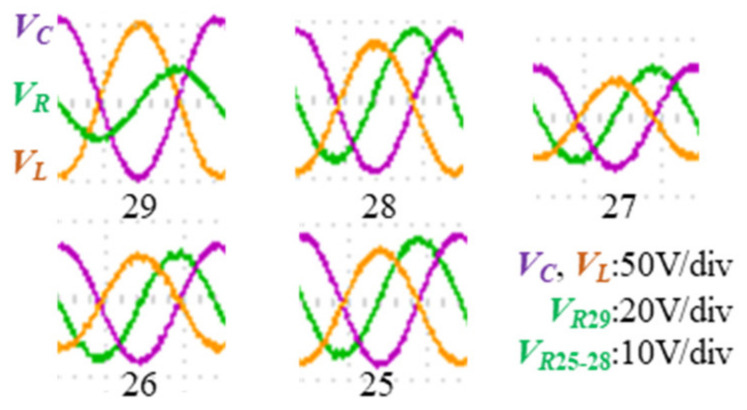
Measurements of the voltages of the inductance, capacitance, and resistance.

The compensated capacitance values and inductance values of magnetic devices are obtained with the impedance tester WK 6500B. The sinusoidal excitation is provided by a signal generator and a power amplifier. The RMS value of the input voltage is 8 V and the RMS value of the loop current is 0.8 A. As shown in Figure 8, the voltages of the inductance, capacitance, and resistance are measured by the oscilloscope.

**Figure 9:**
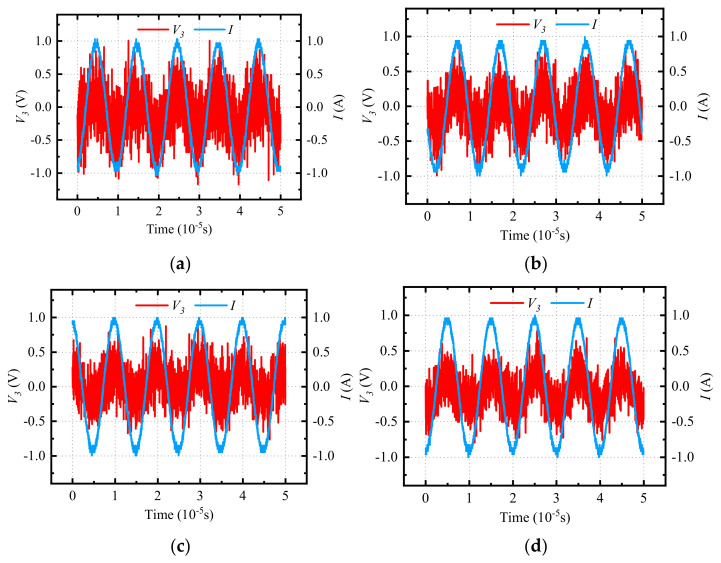

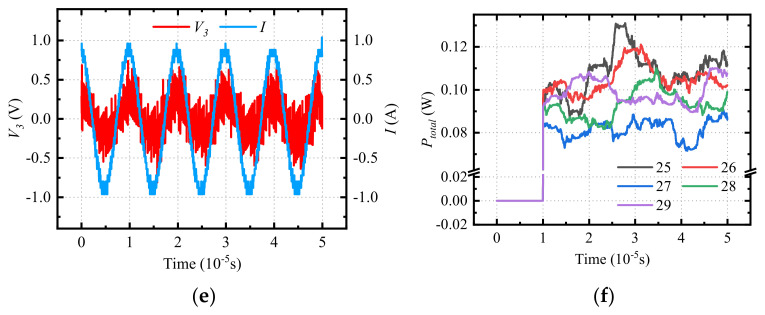
Measurements of virtual voltage, current, and total loss of magnetic devices. (**a**) *V*_3_ and *I* for 25-turn inductor, (**b**) *V*_3_ and *I* for 26-turn inductor, (**c**) *V*_3_ and I for 27-turn inductor, (**d**) *V*_3_ and *I* for 28-turn inductor, (**e**) *V*_3_ and *I* for 29-turn inductor, (**f**) the total loss of all measured devices.

These results are substituted into the analytical equations of the incomplete capacitance compensation method and the total loss of the magnetic device is calculated. The virtual voltage, current, and total loss measurements are shown in Figure 9.

**Figure 10:**
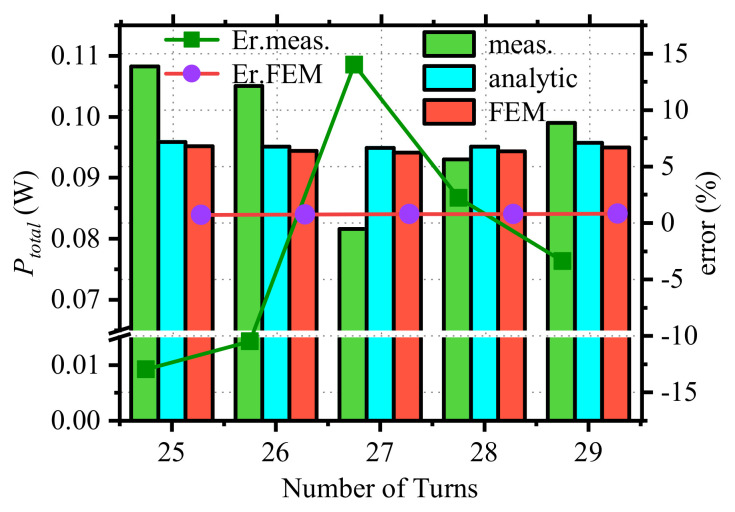
Measurements of the total loss of an optimized designed magnetic device.

The loss measurements in Figure 9e were averaged to give the final total loss value. As shown in Figure 10, the measured, finite element simulated, and analytical values of the total device loss were compared, and the error between the analytical value and them was calculated.

Figure 10 depicts the variation of the total loss with the number of turns of the winding obtained by the proposed analytical method, FEM, and experimental measurements. The total loss obtained by these three methods is represented by ‘analytic’, ‘FEM’, and ‘meas.’, respectively. ‘Er.FEM’ indicates the error between the analytical method and FEM. The error between the experimental results and the analytical method is expressed by ‘Er.meas.’:

As shown in Figure 10, the optimum number of turns obtained by the three methods is 27 turns, and the total loss of the device is minimal. The maximum error between the FEM and the analytical method is 0.82%, and the maximum error between the analytical method and the experimental measurements is 13.99%.

The experimental measurement error primarily stems from two sources: the measuring instrument and the measuring circuit. During the loss measurement process, it is essential to gauge relevant voltage parameters within the circuit. The contact impedance between the measuring probe and the measuring circuit significantly influences measurement accuracy. At higher frequencies, this contact impedance introduces phase errors and voltage noise. Employing RF connectors as sampling connection terminals proves effective in mitigating the impact of contact resistance. Additionally, high-frequency parasitic parameters within the measurement circuit can engender phase errors and voltage noise. The method of incomplete capacitance compensation effectively mitigates the impact of phase errors on experimental results. Within the measuring circuit, high-frequency parasitic capacitance, leakage inductance, and winding equivalent resistance contribute to reducing the measured inductance voltage (*V_L_*) compared to its actual value, thus introducing errors. The measurement error documented in this paper falls within acceptable design tolerances. In conclusion, the FEM and experimental results verify the accuracy of the proposed approximation analysis method in the optimal design of the winding structure and prove the validity of the theory of optimal design of magnetic devices.

## 6. Conclusions

In the realm of miniature systems integrating magnetic components, the mitigation of losses and reduction of device size present significant challenges to system miniaturization. With the aim of mitigating losses in magnetic components, this paper introduces an optimal design methodology tailored for loss-limited magnetic devices. Validation through finite element simulations and experimental findings underscores the efficacy of this approach. The proposed methodology acknowledges the intricate interplay between the loss model of the device and its design parameters and systematically decouples these variables—such as frequency and winding geometric parameters—to derive a closed expression for design optimization. Building upon this foundation, we have formulated an optimization design approach that delineates the design parameters through analytical equations. Comparative with existing optimization methodologies underscores the simplicity and practicality of the proposed approach. Moreover, this paper explores the method’s applicability in optimizing magnetic devices for microsystems, encompassing diverse excitation conditions and conductor types. In the context of miniaturization in microsystem applications, advancing toward more comprehensive and user-friendly optimization techniques for designing loss-limited magnetic devices is the future trajectory.

## Figures and Tables

**Table 1:** Device parameters for conductor diameter optimization design.

Symbol	*m*	*N_v_*	*d* (mm)	*ID* (mm)	*OD* (mm)	*v_s_* (mm)
devices	3	*N*_1_ = 25, *N*_2_ = 20, *N*_3_ = 15	0.1, 0.2, 0.26, 0.3, 0.4, 0.5, 0.6, 0.7, 0.8	9.1	21.1	0.1

**Table 2:** Device parameters for number of turns optimization design.

Symbol	*N_v_*	*h* (mm)	*ID* (mm)	*OD* (mm)
Group 1 devices (100 kHz)	52, 53, 54, 55, 56, 57	7.62	13.34	23.62
Group 2 devices (800 kHz)	28, 29, 30, 31, 32, 33, 34	7.62	13.34	23.62

**Table 3:** Device parameters for winding turns design (experimental).

Symbol	*N_v_*	*d* (mm)	*ID* (mm)	*OD* (mm)	*h* (mm)
Devices (100 kHz)	25, 26, 27, 28, 29	0.65	13.34	23.62	7.62

## Data Availability

Data are contained within the article.
